# Systematic Investigation of LC Miniaturization to Increase Sensitivity in Wide-Target LC-MS-Based Trace Bioanalysis of Small Molecules

**DOI:** 10.3389/fmolb.2022.857505

**Published:** 2022-07-18

**Authors:** Veronika Fitz, Yasin El Abiead, Daniel Berger, Gunda Koellensperger

**Affiliations:** ^1^ Department of Analytical Chemistry, Faculty of Chemistry, University of Vienna, Vienna, Austria; ^2^ Vienna Doctoral School in Chemistry (DoSChem), University of Vienna, Vienna, Austria; ^3^ Vienna Metabolomics Center (VIME), University of Vienna, Vienna, Austria; ^4^ Chemistry Meets Biology, University of Vienna, Vienna, Austria

**Keywords:** miniaturization, chromatography, LC-MS, metabolomics, exposomics, coverage, sensitivity

## Abstract

Covering a wide spectrum of molecules is essential for global metabolome assessment. While metabolomics assays are most frequently carried out in microbore LC-MS analysis, reducing the size of the analytical platform has proven its ability to boost sensitivity for specific -*omics* applications. In this study, we elaborate the impact of LC miniaturization on exploratory small-molecule LC-MS analysis, focusing on chromatographic properties with critical impact on peak picking and statistical analysis. We have assessed a panel of small molecules comprising endogenous metabolites and environmental contaminants covering three flow regimes—analytical, micro-, and nano-flow. Miniaturization to the micro-flow regime yields moderately increased sensitivity as compared to the nano setup, where median sensitivity gains around 80-fold are observed in protein-precipitated blood plasma extract. This gain resulting in higher coverage at low µg/L concentrations is compound dependent. At the same time, the nano-LC-high-resolution mass spectrometry (HRMS) approach reduces the investigated chemical space as a consequence of the trap-and-elute nano-LC platform. Finally, while all three setups show excellent retention time stabilities, rapid gradients jeopardize the peak area repeatability of the nano-LC setup. Micro-LC offers the best compromise between improving signal intensity and metabolome coverage, despite the fact that only incremental gains can be achieved. Hence, we recommend using micro-LC for wide-target small-molecule trace bioanalysis and global metabolomics of abundant samples.

## 1 Introduction

The physicochemical diversity and wide concentration ranges of metabolites in biological samples to date prevent comprehensive coverage of the metabolome by a single (or even a few) analytical methods. Methods based on liquid chromatography coupled to mass spectrometry (LC-MS) offer the best sensitivity, highest versatility regarding physicochemical coverage and dynamic ranges between two and four orders of magnitude. Specifically, chromatographic separation supports the identification of isomers, reduces ion suppression and improves detection of low-abundant compounds (Lu et al., 2017; Alseekh et al., 2021). LC-MS-based metabolomics experiments are most frequently carried out in microbore scale (i.e., 1.5–3.2 mm inner column diameter and flow rates of 100–500 μl/min) (Vasconcelos Soares Maciel et al., 2020). Microbore systems are robust and convenient to use, accommodate short and steep gradients and provide high-performance chromatography with peak widths around 3 s (full width at half maximum). The workflows established for high resolution mass spectrometry (HRMS)-based analysis depend on highly repeatable features with regard to retention time and signal intensity. Microbore LC systems allow robust plug-and-play operation while providing reproducible retention times, peak shapes and signal intensities, supporting automated data processing in the course of non-targeted experiments. Likewise, narrow peak shape, technical reproducibility of signal intensities, minimal system carryover and linear detector response build the basis for (relative and absolute) quantification.

In contrast to small-molecule -*omics*, proteomics and peptide LC-MS-analyses are commonly carried out on the nano-scale (i.e., 10–150 µm column i.d. and flow rates of 0.1–1 μl/min). Gradients in proteomics and peptide analysis are typically much longer (in the order of an hour) and eluent composition covers a narrower span of organic eluent content. Nano-LC coupled to nano-ESI-MS offers unrivalled mass sensitivity essential for the analysis of low-volume samples. The assets but also challenges of nano-LC are related to the low flow rates employed. On the one hand, sensitivity can be vastly increased by reduced on-column sample dilution and compatibility with nano-ESI, offering itself unique benefits for ionization (Juraschek et al., 1999; Schmidt et al., 2003; Kourtchev et al., 2020). On the other hand, the low flow rates and small column dimensions make the whole system more susceptible to void volumes, clogging of column and capillaries/emitter, mass overload and associated system carryover, etc. (Noga et al., 2007). In principle, these stressors also affect microbore LC, but are more pronounced at the very low flow rates of nano-LC, and complicate successful handling in practice. Hence, nano-LC is not as widely established in small-molecule -*omics* as it is in proteomics, but it has been successfully applied for (xeno-)metabolomics analysis especially for cases where low available sample volumes demanded the smallest possible analysis platform (Lanckmans et al., 2006; Nakatani et al., 2020; Geller et al., 2022). In fact, LC miniaturization for small-molecule analysis is iteratively discussed in literature as a means of increasing sensitivity when dealing with low sample amounts (Chetwynd and David, 2018; Nakatani et al., 2020; Sanders and Edwards, 2020).

While analytical flow and nano-flow LC-MS platforms are routinely used in metabolomics and proteomics analyses (Shi et al., 2004; Wilson et al., 2015; Yi et al., 2017), the interest for micro-flow platforms is increasing in both research communities as a means to enhance sensitivity and save analysis cost (coming from analytical flow) (Greco et al., n.d.; Gray et al., 2016; Cebo et al., 2020; King et al., 2020) or to enhance robustness and reproducibility of the analysis (coming from nano-flow) (Bian et al., 2020). LC miniaturization maximizes signal intensities from a given amount of injected sample and potentially extends the analysis scope toward molecules with low abundance or detector response. Signal intensity is key for compound identification as signal intensity thresholds determine the triggering, acquisition and quality of MS/MS spectra.

A handful of studies have compared the performance of specifically optimized miniaturized LC-MS platforms with their established microbore LC-MS workflows for metabolomics or other multi-residue small-molecule analyses (Chetwynd et al., 2014; Nakatani et al., 2020; Zardini Buzatto et al., 2020; Geller et al., 2022). With the present study, we address the following question: Assuming that sample volume is not a limiting factor, would LC miniaturization allow to broaden the analyte scope by extending coverage toward low abundant analytes? Injecting the same sample volume on a smaller analytical platform equals a large volume injection, which is successfully applied for, e.g., proteomics or environmental analysis, but without increasing the actual amount of injected sample–a considerable asset for metabolomics experiments since they usually deal with dense sample matrices and long sequence runs. LC miniaturization holds the potential for maximizing sensitivity without the cost of polluting ion source and mass spectrometer with additional sample. Here, we pinpoint the benefit and challenges of LC miniaturization for non-targeted multicomponent small molecule analysis in practice by transferring a typical metabolomics method from analytical to micro- and nano-flow regime while holding injection volume, mobile and stationary phases, gradient and detection parameters constant.

## 2 Experimental

We compared a standard analytical scale (250 μl/min) reversed-phase metabolomics method with two grades of miniaturization, micro- (57 μl/min) and nano-flow (0.3 μl/min) (Vasconcelos Soares Maciel et al., 2020), by injecting a series of standards and matrix samples on each platform. We analyzed spiked exogenous compounds at different concentrations and endogenous human plasma metabolites at natural abundance levels. The analytes were selected to cover a wide range of physicochemical properties and show different grades of reversed phase retention (see Figure 6) to monitor chromatographic enrichment and retention-related impacts on signal intensity.

### 2.1 Standards and Solvents

Acetonitrile (ACN) and water were of LC-MS grade and ordered at Sigma-Aldrich (Vienna, Austria) and Fisher Scientific (Vienna, Austria). Formic acid ≥99% and methanol (MeOH) were also of LC-MS purity and ordered at VWR International (Vienna, Austria).

The mycotoxins aflatoxin B1 and G2, ochratoxin A, sterigmatocystin, T2-toxin and zearalenone were obtained from RomerLabs (Tulln, Austria). Aflatoxin M1, aflatoxicol, alternariol, and ochratoxin alpha were obtained from Toronto Research Chemicals (Ontario, Canada). A total of 10 mycotoxins were analyzed. Pharmaceutical and agrochemical standards were kindly provided by Eurofins Umwelt Österreich GmbH & Co KG (39 and 9 compounds, respectively). Standards were obtained in dissolved form or weighed and dissolved in appropriate solvent to obtain single stock solutions. All standards were of HPLC-grade or LC-MS-grade purity. Molecules and sum formulas are listed in Supplementary Table S1.

In the following, we compare peak width and peak shape, repeatability of detector response, retention time stability, signal intensity and peak concentration for model molecules that are detected in all three setups in spiked plasma extract at a concentration of 1 μg/L, except ceftiofur and coumaphos, which are assessed at 10 μg/L. Linear range, sensitivity, matrix effect and limit of detection are assessed and compared for molecules with a linear relation of concentration and detector response in all three setups. Coverage and signal intensity ratios are additionally assessed using a panel of molecules detected in plasma extract at naturally occurring abundances (48 endogenous metabolites, 3 xenobiotics). Details are listed in Supplementary Tables S2–S5.

### 2.2 Spiking Solutions

Single stocks were stored at −20°C until they were volumetrically combined to give two multicomponent mixtures: one containing pharmaceuticals/agrochemicals and one containing mycotoxins. For both mixtures, single stocks were combined volumetrically and evaporated to dryness in a vacuum centrifuge at room temperature. The pharmaceutical/agrochemical residue was reconstituted in MeOH to give a multicomponent standard with a concentration of 50,000 μg/L. Further 1:10 dilution steps with MeOH yielded multicomponent standards with concentrations of 5,000, 500, 50, 5 and 0.5 μg/L. The mycotoxin residue was thoroughly reconstituted in 5% (v/v) ACN to give a multicomponent standard with a concentration of 500 μg/L. Further 1:10 dilution steps with 5% (v/v) ACN yielded multicomponent standards with concentrations of 50 and 5 μg/L.

### 2.3 Plasma Extraction

Pooled human blood plasma (two donors) was purchased in frozen form (dry ice) at Innovative Research, Inc. (46430 Peary Court, Novi, Michigan, United States) and stored at −20°C until sample preparation. After thawing at room temperature, 2 ml of plasma was transferred to a 15 ml Falcon tube and mixed with 6 ml acidified ACN (ACN +0.1% (v/v) formic acid). The mixture was vortexed for 3 min and kept at −20°C for 1 h to allow protein precipitation, then it was vortexed again and mildly centrifuged for 5 min at 2,000 rcf. The supernatant was collected and transferred to Eppendorf tubes for high-speed centrifugation (14,000 rcf, 10 min). Centrifugation steps were performed at room temperature on a HERMLE Z446K centrifuge. The supernatants were carefully aspirated and mingled in 5 ml Eppendorf tubes. Seven aliquots of 400 µl each were prepared in brown 1.5 ml HPLC glass vials with screw caps and septum.

### 2.4 Samples

Experiments were based on spiked plasma extract (matrix samples) and spiked neat solvent. For neat solvent samples, 2 ml of LC-MS-grade water were taken through the same sample preparation procedure as described for plasma extracts (Section 2.3). Next, 400 µl aliquots of plasma extract or solvent were spiked with the previously prepared multicomponent mixtures (pharmaceuticals/agrochemicals and mycotoxins, respectively), giving six concentration levels (5,000, 1,000, 100, 10, 1, and 0.1 μg/L) plus one zero sample for each of the two matrices. Mycotoxins were not spiked to samples of the highest concentration and were diluted 1:10 in all other concentration levels as compared with the spiked pharmaceuticals/agrochemicals, giving sample concentrations of 100, 10, 1, 0.1, and 0.01 μg/L plus zero sample. Spiked samples were evaporated to dryness, thoroughly reconstituted in 5% (v/v) ACN and transferred to 1.5 ml brown-glass HPLC-vials with 200 µL glass inserts and screw caps with slit septum for analysis. Portions of sample that were not needed at the moment were kept in their original HPLC-vials and stored at −20°C in dissolved form.

### 2.5 Instrumental Setups

Three analytical setups were compared in this analysis. All were based on C18 HSS T3 column chemistry with acidified H_2_O/ACN as eluent system, and on detection *via* HRMS with a Q Exactive HF quadrupole-Orbitrap mass spectrometer (Thermo Scientific). The microplatform was stringently scaled to maintain the same linear flow velocity as in the analytical flow equivalent. The three platforms employed the same sub-2µm stationary material and were operated with volumetric flow-rates close to their van Deemter optima.

Owing to the great structural diversity of metabolites, it is impossible to assess the entire metabolome with one analytical platform (Patti, 2011; Lu et al., 2017). Nevertheless, the aim is to capture as many molecules as possible and separations in global metabolomics are not tailored to specific molecules but most frequently use generic LC gradients spanning very low to very high organic eluent content and medium run times. Fully wettable stationary phases tolerate 100% (v/v) aqueous eluent composition and offer retention for the more polar analytes that would be flushed away with minimal organic solvent in the eluent compared with conventional C18 phases. Next to analyte enrichment, several other parameters along the analytical process influence the intensity of the resulting detector signal: Matrix density, dilution, solvent and volume of the injected sample, extra-column volumes and flow-rate, amount and mass loadability of the stationary phase, ionization efficiency depending on analyte chemistry, eluent, coeluting matrix, droplet size related to emitter geometry, spray voltage; and finally, ion transfer, width of *m/z* scan window, ion suppression effects in the c-trap and detection efficiency in the mass analyzer. All of these parameters should be adapted to the type of sample and analytes of interest and should finally suit the analytical platform to achieve the highest possible sensitivity. Optimization of the whole analytical procedure is indeed quite specific for each application. With this study, we want to elaborate the sensitivity potential enabled by LC miniaturization, i.e., reduced column inner diameter and reduced flow-rate, for wide-target small-molecule analysis. It serves comparability to keep as many of the parameters constant as possible (injection volume, mass spectrometer, detection parameters) and adapt only parameters that are directly related to the flow rate (LC instrument, flow rate, ion source parameters). For the adapted parameters, we followed vendor recommendations as far as possible to ensure we operated each instrumental platform under the respective optimal conditions while offering suitable conditions for a wide variety of analytes.

An overview of the key method features is given in Table 1. Further details can be found in the text below.

**TABLE 1:** Key features of the three analytical setups.

	Analytical setup	Micro-setup	Nano-setup
LC instrument	Vanquish Duo UHPLC	Vanquish Duo UHPLC	UltiMate 3000 RSLCnano
Column i.d.	2.1 mm	1.0 mm	0.075 mm (separation)
			0.3 mm (trap)
Flow rate	250 μL/min	57 μL/min	Separation: 0.3 μL/min
			Loading: 30 μL/min
Inject. volume	3 µL	3 µL	3 µL
ESI source	Ion Max with HESI-II-probe	Ion Max with HESI-II-probe	Nanospray Flex
Emitter i.d.	100 µm	50 µm	30 µm
Spray voltage	3.5 kV	3.5 kV	1.9 kV
Other source parameters	Flow rate default for temperatures and gas flows	Flow rate default for temperatures and gas flows	Manually adjusted emitter position

#### 2.5.1 Analytical Flow Setup

The standard LC-setup was built upon a Vanquish Duo UHPLC system (Thermo Scientific) consisting of a solvent rack, two binary pumps, a split sampler with two injection valves, and a column compartment. The capillary setup was optimized for analytical flow regimes and consisted of 100 µm i.d. Viper-capillaries (Thermo Fisher Scientific) pre- and post-column. An Acquity UPLC HSS T3 column (2.1 mm i.d. × 150 mm, 100 Å, 1.8 µm, Waters) equipped with a VanGuard Pre-Column (2.1 mm i.d. × 5 mm, Waters) was eluted in gradient-mode with a flow rate of 250 μl/min at 35°C. Mobile phase A was H_2_O + 0.1% (v/v) formic acid, mobile phase B was ACN +0.1% (v/v) formic acid. The following gradient was applied: 0–1 min 1% B, 1–5 min ramp to 50% B, 5–12 min ramp to 99% B, 12–15 min hold at 99% B, at 15 min switch to 1% B, followed by 15–22 min re-equilibration at 1% B. The injection volume was 3 µl and the injector needle was washed with 80% ACN for 15 s after each injection. The column was connected to an Ion Max Source with a Heated Electrospray Ionization (HESI-II) Probe and a 100 µm i.d. stainless steel emitter (Thermo Fisher Scientific) via a 100 µm i.d. Viper-capillary, a zero dead-volume grounding union, and a piece of 100 µm i.d. PEEK-capillary.

#### 2.5.2 Micro-Flow Setup

This LC-setup was built upon the same Vanquish Duo UHPLC system (Thermo Scientific) as the analytical flow setup. An Acquity UPLC HSS T3 column (1 mm i.d. × 150 mm, 100 Å, 1.8 µm, Waters) equipped with a VanGuard Pre-Column (2.1 mm i.d. × 5 mm, Waters) was eluted in gradient-mode. The flow rate was volumetrically scaled to maintain the same linear flow velocity as in the analytical setup and was held constant at 57 μl/min. Column temperature, eluents, and gradient were the same as described for the analytical setup. The same 100 µm i.d. Viper capillaries were used pre-column as described above, which led to slight gradient delay in combination with the lower flow rate. It was necessary to prolong the re-equilibration step to 9 min, resulting in a total runtime of 24 min: 0–1 min 1% B, 1–5 min ramp to 50% B, 5–12 min ramp to 99% B, 12–15 min hold at 99% B, at 15 min switch to 1% B, followed by 15–24 min re-equilibration at 1% B. Injection volume, needle wash and column temperature were the same as in the analytical flow setup. To avoid post-column peak broadening, the post-column flow path was adapted to the lower volumetric flow rate: The column was connected to an Ion Max Source with a Heated Electrospray Ionization (HESI-II) Probe *via* a 50 µm i.d. × 350 mm nanoViper-capillary, a zero dead-volume grounding union, and 50 µm i.d. × 150 mm nanoViper-capillary. The ion source was equipped with a 50 µm i.d. stainless steel emitter. Flow-path adaptations were made according to (Greco et al., n.d.).

#### 2.5.3 Nano-Flow Setup

For the nano-flow setup, a trap-and-elute configuration was chosen to increase loading capacity and loading flow rate. An UltiMate 3000 RSLCnano system (Thermo Scientific) consisting of SRD-3400 solvent rack, NCS-3500RS pump module containing the column compartment and an 850 bar 10-port switching valve, and a WPS-3000TPL RS temperature-controlled autosampler equipped with a 350-bar 8-port-valve and an 850-bar injection valve. Fluidic setup and capillary dimensions followed vendor recommendations to minimize pre-column extra-column volumes. Micro-flow (30 μL/min) was delivered by a ternary micro pump for preconcentrating the sample on a trapping column. The loading pump delivered the flow through the autosampler injection valve via the 10-port switching valve in the column compartment onto the trapping column. Nano-flow (0.3 μL/min) was delivered by a nano/capillary pump and directed onto the nano-column. Flow rate was regulated with an integrated ProFlow flowmeter. The nano/capillary pump delivered the flow to the nano-column *via* the 10-port switching valve in the column compartment. A 3 µL sample plug was drawn in microliter-pickup mode through a 2.4 µL injection needle and into a 20 µL sample loop. LC-MS-grade water with 0.1% (v/v) formic acid served as pickup-fluid. The sample was injected into the loading-flow path and accumulated on the trapping column for 1 min. The trapping column effluent was directed to waste during the loading procedure. Subsequent analysis was carried out in back-flush mode, i.e., the trapping column was switched in line with the nano-column by rotating the column compartment 10-port valve, now carrying the entire nano/capillary-pump-gradient through the trapping column in reversed direction and through the nano-column to the MS. Pre-concentration setups with commercial equipment typically employ the same stationary phase chemistry for trap column and analytical column (Wilson et al., 2015), but with larger particle size and hence less retentivity of the trap column. A nanoEase *M/Z* HSS T3 trap column (0.3 × 50 mm, 100Å, 5 μm, Waters) was used for pre-concentration and a nanoEase *M/Z* HSS T3 nano-column (0.075 × 150 mm, 100Å, 1.8 µm, Waters) for analyte separation. The column compartment was kept at 35°C.

Eluent composition was the same for trapping and separation: Eluent A was H_2_O + 0.1% (v/v) formic acid and eluent B was ACN +0.1% (v/v) formic acid. Trapping was pursued for 1 minute with 0% B (isocratic). While the aliphatic groups of ordinary C18 material collapse in 100% aqueous environment, the HSS T3 chemistry is fully wettable. We chose this material to allow a loading step without organic modifier to retain polar compounds as far as possible. The trapping column was switched in line with the nano-column 1 min after injection and with another 1-min delay, a gradient from 1 to 99% B in 11 min was delivered by the nano/capillary-pump for separation on the nano-column. The gradient was followed by a 6-min flush with 99% B and 23 min re-equilibration at 1% B. The nano-column was connected to a nano-ESI source with a piece of 20 µm i.d./280 µm o.d. fused silica tubing, a PTFE sleeve and a zero dead volume PEEK union. Considering the complexity of samples obtained by non-selective liquid-liquid-extraction and centrifugation, a stainless-steel emitter with an i.d. of 30 µm was chosen to ensure longer durability and avoid clogging as compared to silica emitters with lower inner diameter. Emitter position was adjusted manually. Spray voltage was 1.9 kV in positive ionization mode.

#### 2.5.4 Mass Spectrometry

HRMS was performed with a Q Exactive HF quadrupole-Orbitrap mass spectrometer (Thermo Scientific). The following parameters were used for all three setups: MS1 spectra (profile mode), scan range 80–1200 *m/z*, positive polarity, resolution 120,000, AGC target 3e6, maximum injection time 200 ms, and S-lens RF-level 50. For ionization, two different electrospray sources were used: A Nanospray Flex ion source equipped with a 30 µm i.d. steel emitter for the nano-scale setup, and an Ion Max source equipped with a HESI-II-probe and steel emitter for micro- and analytical setup. Both sources were purchased from Thermo Fisher Scientific. Emitter i.d. was 100 µm for the analytical setup, while the micro-setup required a reduced emitter i.d. of 50 µm. We optimized the ESI parameters and carried out the experiments under optimum condition for the respective flow regime. Spray voltage was 1.9 kV for the nano-setup and 3.5 kV for micro- and analytical setup, respectively. Flow rate sensitive parameters for HESI-ionization were adapted according to vendor recommendations: for micro-setup (57 μL/min), capillary temperature was 250°C, sheath gas 30.70, auxiliary gas 10.00, spare gas 1.00, and probe heater temperature 157°C. For analytical setup (250 μL/min), capillary temperature was 253.13°C, sheath gas 46.25, auxiliary gas 10.63, spare gas 2.13, and probe heater temperature 406.25°C.

### 2.6 Data Evaluation

After data acquisition, vendor-specific profile mode files were centroided with the msConvert GUI application (version 3.0.19014-f9d5b8a3b) from the ProteoWizard Toolkit applying the peakPicking-filter (vendor msLevel = 1-1) and mzML as output format (Chambers et al., 2012). Centroided data were subjected to targeted data evaluation in Skyline (Adams et al., 2020). A mass extraction window of 10 ppm was used to generate extracted ion chromatograms of the target compounds. The chosen procedure outweighed calibration-related differences in mass accuracy between the datasets and avoided loss of peak area in the *m/zm/z* dimension for all three setups (Vereyken et al., 2019). Evaluation of chromatographic parameters focused on [M + H]^+^ adducts of the monoisotopic peaks. For selected compounds, extracted ion chromatograms were generated based on [M]^+^, [M + NH_4_]^+^ or [M + H-H_2_O]^+^ adducts. For spiked exogenous compounds, area values of monoisotopic EICs were used to characterize signal intensity, signal reproducibility, matrix effect, slope and linear range of calibration curves, and limit of detection. Furthermore, we assessed chromatographic peak width and symmetry, retention time stability and peak concentration. Signals with less than three consecutive data points per peak were dismissed. Since some compounds showed background noise, signals below 2× the averaged signal of a matrix-specific zero sample (solvent or plasma extract with a spiked concentration of 0 μg/L, *n* = 4) was put in place as additional filter. Formulas for the calculation of the parameters can be found in the results section.

The same method characteristics were assessed for a panel of metabolites detected in non-spiked plasma extract, except for matrix effect, calibration curve and LOD. Peak concentration was expressed relative to the analytical flow setup. The chosen metabolites represent major metabolite groups found in the human serum metabolome database (Psychogios et al., 2011): Small organic acids, nucleobases, steroid hormones, sugar phosphates, amino acids, and lysophospholipids. The molecules were identified by retention time comparison with authentic standards or literature as noted in Supplementary Table S1.

## 3 Results

This study elaborates the impact of LC miniaturization in small-molecule LC-MS analysis. We compared two miniaturized platforms with different grades of miniaturization, micro- and nano-flow, with the standard analytical flow platform, for depicting the metabolome of an abundant sample. The platforms were characterized by performance parameters critical for current practices of non-target bioanalysis-like peak picking and statistical analysis, emphasizing sensitivity and metabolome coverage. We compared analytical figures of merit for model molecules that were detected in all three setups in spiked plasma extract at a concentration of 1 μg/L, except ceftiofur and coumaphos, which were assessed at 10 μg/L, or for molecules that showed a linear relation of signal intensity and concentration in all three setups (linear range, sensitivity, matrix effect, limit of detection), respectively. Coverage and signal intensity ratios were additionally assessed using a panel of endogenous metabolites detected in plasma extract at naturally occurring abundances. We analyzed a panel of molecules with wide chemical diversity with logP values between −4.4 and 7.7 and molecular weights spanning approximately 85–550 Da. The results are listed in Supplementary Tables S2–S5.

Non-targeted experiments should cover both ionization polarities since some metabolite classes ionize more effectively as anions (constituents of the central energy metabolism like small organic acids, sugars and their di-/triphosphates, etc.) and others as cations (amino acids, nucleobases, nucleosides and nucleotides, steroids, several lipid classes, etc.). Acquiring positive and negative mode data in an automated fashion or even in one chromatographic run is desirable. In particular, the Orbitrap mass analyzer supports fast polarity switching, which allows to record positive and negative ionization mode data near-simultaneously and offers a substantial increase in analysis throughput. The used nanoESI source, however, did not allow fast polarity switching. The emitter position needs to be adjusted for each ionization mode manually, precluding automated acquisition of positive and negative mode data in one run. Additionally, the nanoESI spray is less stable in negative mode especially for eluent compositions with a high aqueous content, as observed by us and others (Nguyen-Khuong et al., 2018). For our experiments we therefore used positive ionization mode. The described chromatographic phenomena apply to both polarities. When interpreting our results it should be kept in mind that ionization efficiency and matrix effect can differ in negative ionization mode.

### 3.1 Chromatographic Quality, Repeatability, and Peak Shape

#### 3.1.1 Peak Width and Symmetry

**FIGURE 1:**
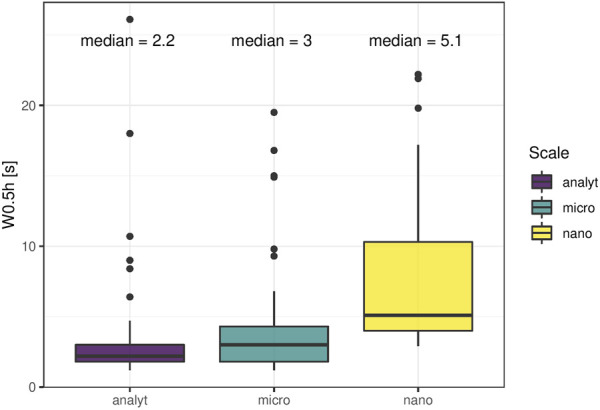
Peak width. Full width at half maximum was assessed for 53 molecules comprising endogenous (ceftiofur and coumaphos 10 μg/L, caffeine, paraxanthine and theobromine at naturally occurring abundance; all others 1 μg/L) molecules in plasma extract. Lysophospholipids were broader compared to the rest in all setups. Further, thiophosphate, sulfonamide/sulfonate and nitrate-containing compounds had distorted peak shapes in the nano setup.

Peak width (here: width at half-maximum) and peak symmetry are related to chromatographic resolution and enrichment success. Narrow peaks ensure maximal separation efficiency and signal intensity, resulting in cleaner MS/MS spectra and better detection limits. Peak width homogeneity affects the quality of fragment spectra as the average peak width is set for data-dependent MS/MS acquisition (average peak width for dynamic exclusion and apex trigger). It also influences the quality of peak picking by commonly applied software like XCMS, where a window of peak widths needs to be defined to help differentiate chromatographic peaks from background signals. Miniaturization to modular LC-systems holds the risk of peak broadening because the ratio of column-volume and flow rate to extra-column volumes tends to be less favorable compared to columns with a greater inner diameter even with optimized flow-path connections. Median peak width was comparable between the setups (Figure 1). Phospholipids eluted as broader peaks on all platforms with median peak widths around 5 s under micro- and analytical flow, and around 7 s under nano-flow conditions. Sn-1 and sn-2 positional isomers were fully separated in the former two setups but were not baseline separated in the nano-platform. Thiophosphates (acephate, coumaphos, methamidophos), sulfonamides (sulfachlorpyridazine, sulfadiazine, sulfadimethoxine, sulfamethazine, sulfamethoxazole, sulfamethoxypyridazine, sulfaquinoxaline, sulfathiazole) and the sulfonate florfenicol, as well as several nitrates-containing compounds (dimetridazole, dinoterb, furazolidone, ronidazole) eluted as very broad peaks in the nano-setup, while the same compounds exhibited excellent peak shapes in the micro- and analytical flow regime. Since the same was observed for direct injection mode without enrichment column (data not shown), we assume that the pronounced compound-class specific peak shape distortion in the nano-setup is linked to surface interactions. Unwanted surface interactions are enhanced in nano-LC systems, leading to unexpected chromatographic effects even for compounds with otherwise good retention, which complicates the choice of optimal peak width for data-dependent MS/MS acquisition and non-targeted peak picking. Additionally, most molecules displayed compound-specific tailing in the nano-setup at all tested concentrations. Analytical and micro-platform, on the other hand, showed almost perfect peak symmetry. Tailing peaks reduce chromatographic resolution and hold the risk of masking low abundant analytes through ion suppression. Overlapping peaks lead to chimeric spectra during fragmentation, undermining the accuracy of compound identification.

#### 3.1.2 Signal Stability

**FIGURE 2:**
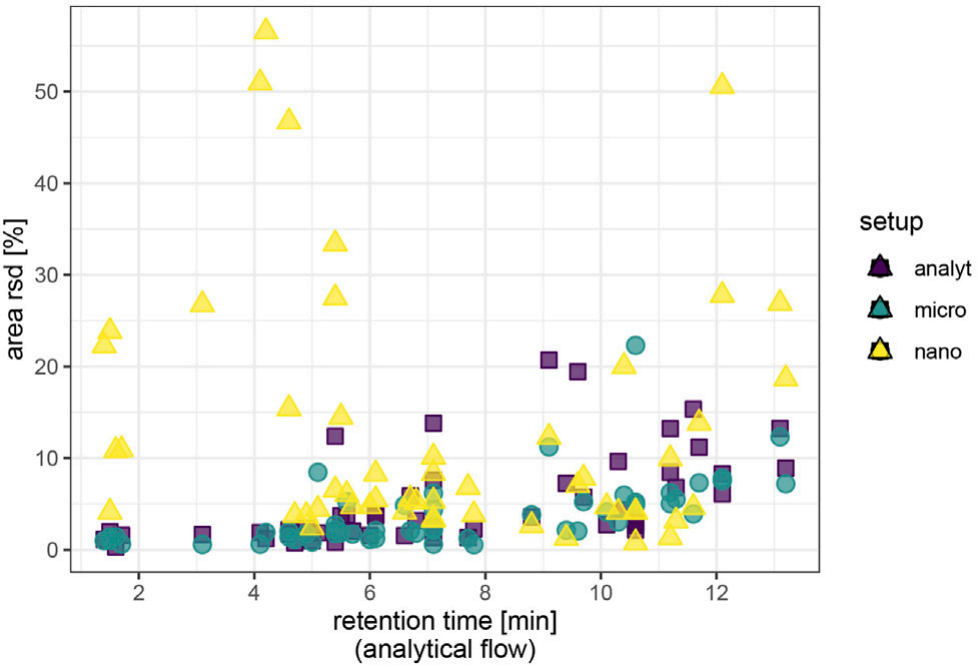
Repeatability of signal intensity based on repeated injections (N = 4) of spiked plasma extract. Endogenous metabolites and caffeine/caffeine metabolites were assessed at natural abundance; ceftiofur and coumaphos at 10 μg/L, all other exogenous compounds at 1 μg/L. Area values are background corrected.

Among the molecules that were detected in all setups, the median repeatability of area values was around 3.7% relative standard deviation in the analytical flow setup for the spiked exogenous test molecules and excellent 2.3% for the endogenous molecules investigated. Repeatability improved upon miniaturization for well-retained exogenous compounds with previously low signal intensity. We observed a decrease of area repeatability with retention time in the analytical flow regime and to a lesser extent in the micro-flow regime (Figure 2). The nano-flow platform showed elevated but satisfactory area repeatability for most of the exogenous compounds. Sulfonamides and florfenicol (sulfonate), nitrates, and thiophosphonates-displayed reduced area repeatability. Notably, the nano-setup displayed optimum repeatability only for a specific retention segment, while the more hydrophilic metabolites (amino acids) were less reproducible due to suboptimal chromatographic enrichment in the trap-and-elute configuration and area rsd had a tendency to increase for the very lipophilic metabolites (lysophospholipids) due to spray destabilization at high proportions of organic solvent in the eluent. Additionally, repeatability did not improve analogously with signal intensity in the nano-flow setup. It is difficult to maintain a stable electrospray throughout the wide gradient with only one spray voltage setting and the delicate stability of the nanospray is even more affected by rapidly changing eluent conditions.

#### 3.1.3 Retention Time Stability

**FIGURE 3:**
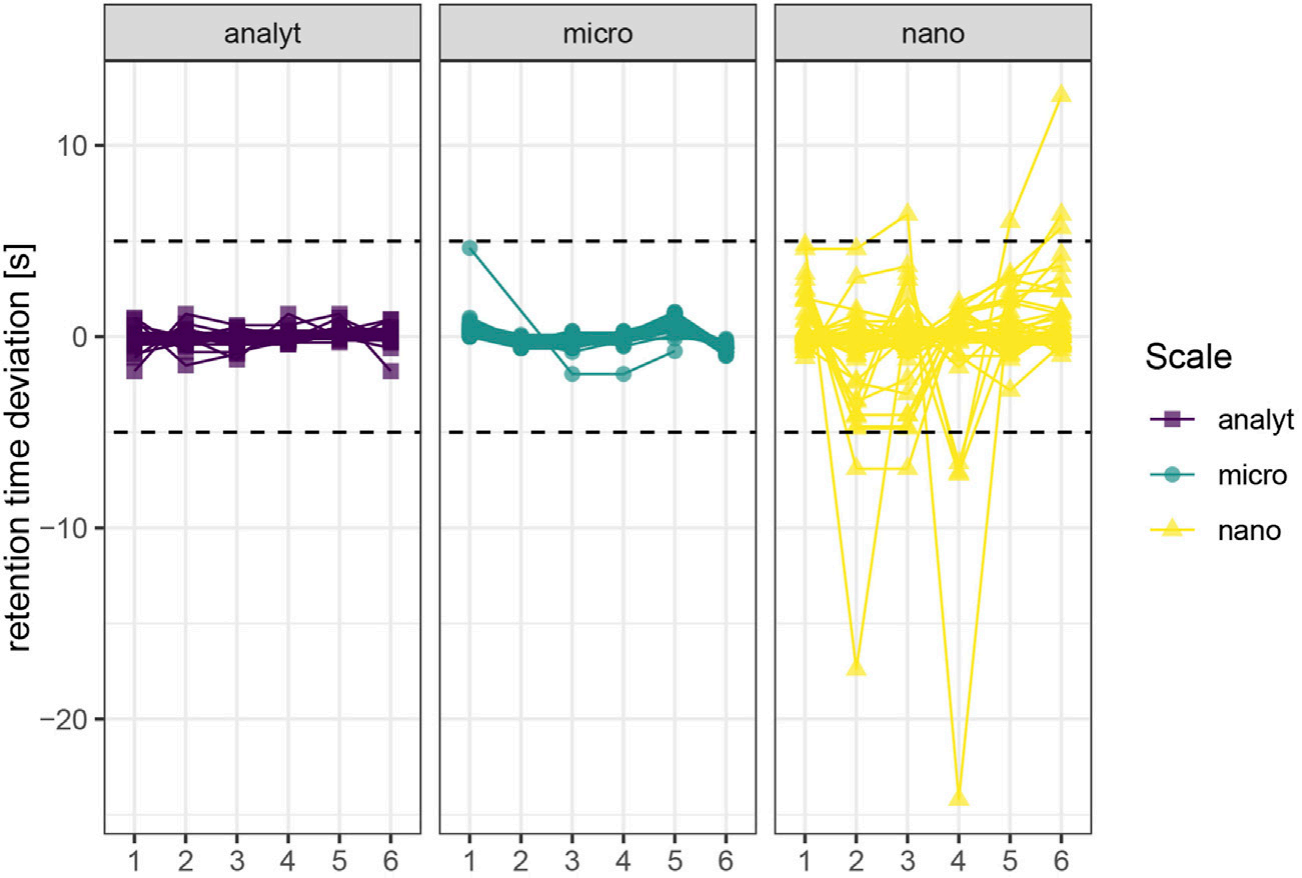
Retention time stability. A quality control sample (c = 1 μg/L) was injceted six times (*n* = 6) spanning 8.5 h (analytical), 9 h (micro) and 15 h (nano). Replicates 4–6 were injected right after another. The dashed line marks 5 s deviation from the mean retention time. Molecules with distorted peak shapes also display reduced retention time stability due to imprecise automatic detection of the peak apex.

We assessed retention time stability based on repeated injections of a quality control sample throughout the injection sequence (c = 1 μg/L, *n* = 6) spanning 8.5 h (analytical), 9 h (micro) and 15 h (nano). Retention times were adequately stable for all three investigated setups, as retention times deviated from the mean less than 5 s during 23 injections (Figure 3). Retention time stability of the nano-setup was comparable to micro- and analytical flow regime, even though absolute retention times were around twice as high due to pronounced gradient delay and resulting duration of the method. However, compounds that eluted as broad peaks (sulfonamides/sulfonate, nitrates, thiophosphates) also displayed reduced retention time repeatability.

### 3.2 Sensitivity

#### 3.2.1 Signal Intensity/Sensitivity

**FIGURE 4:**
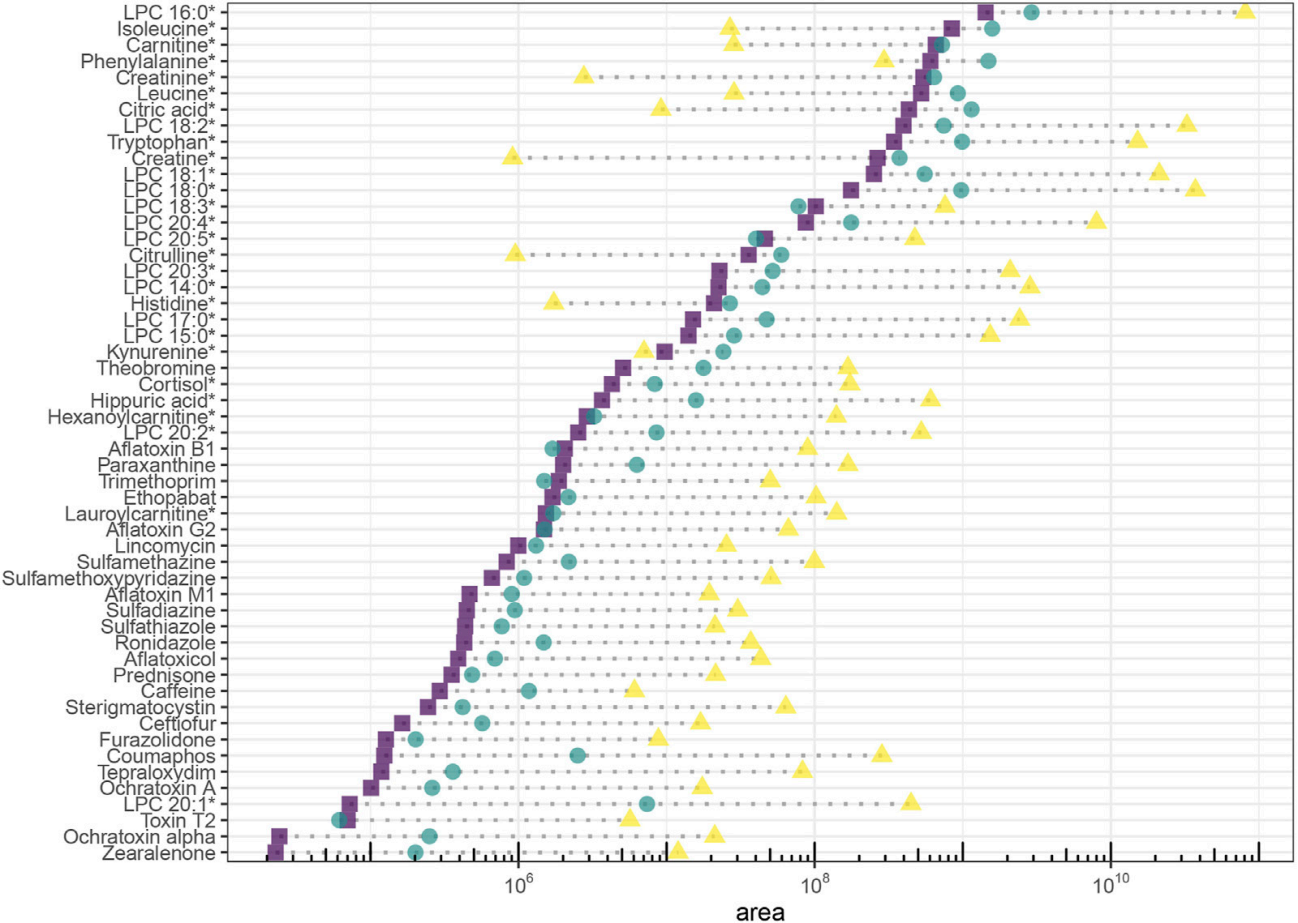
Signal intensity of endogenous (natural abundance) and exogenous molecules (ceftiofur and coumaphos 10 μg/L, caffeine, paraxanthine and theobromine naturally occuring abundance, all others 1 μg/L) in plasma extract. Areas are background corrected. Endogenous molecules are marked with an asterisc.

Yielding the highest signal intensity out of a given amount of sample is the principal goal of LC miniaturization. The assumption for our study is that abundant sample material is available and sample volume is not a limiting factor for sensitivity (e.g., plasma analysis of adult humans). Hence, we injected the same sample volume on each platform. Signal intensity was assessed for all molecules, sensitivity (expressed as slope of calibration curves in linear range) was additionally reported for the spiked exogenous compounds. On average, signal intensity and sensitivity were improved through both miniaturized setups. Area ratios increased with retention time in the nano-setup, underlining that chromatographic enrichment was an important factor to maximize sensitivity. For the micro-setup, this relation was not as straightforward. The actual extent of intensity increase depended on the specific molecule in both setups (Figure 4). Using the micro-flow setup we observed a median increase of signal intensity of around 2-fold for spiked plasma extract, with individual signal intensity ratios ranging between 0.7 and 20 (excluding LPC 20:1, which increased to 100-fold due to very low signal intensity in the analytical flow setup). Downscaling to nano-flow multiplied signal intensities compared to the analytical flow regime: a median 45-fold for the investigated endogenous metabolites and around 75-fold for exogenous molecules. Signal intensity ratios of the more polar compounds fell below 30-fold increase, while individual rather lipophilic molecules exceeded 1,000-fold increase.

#### 3.2.2 Peak Concentration

Volumetric flow rate of the analytical setup (250 μL/min) had been scaled to the smaller column dimensions of the micro-setup (1 mm i.d. vs. 2.1 mm i.d.) to maintain approximately the same linear flow velocity (Eqs 1, 2). Under the assumption that peak width in the miniaturized setups is as narrow as under analytical flow regime, the analyte band is more concentrated and signal intensities theoretically increase by a factor of 4.4 after injecting the same amount of sample. Likewise, the theoretical increase in signal intensity using the nano-setup is 880-fold. However, transferring the whole analytical platform to a lower flow regime opens a multitude of factors that can influence actually obtainable signal intensities. First, chromatographic effects like peak broadening and tailing upon miniaturization can hamper signal intensity and signal-to-noise ratios; second, the different flow rates and peak concentrations can impact ionization efficiency, ion transmission and collection, and Orbitrap analysis. We compared peak concentrations (Eq. 3) and found that (mild) peak broadening affected the concentration of the analyte band in the miniaturized setups. Broader peaks elute in a higher volume of eluent and the concentration reaching the detector is therefore reduced. The theoretical gain in peak concentration as described above is therefore not reached in practice. While both, peak concentration gain and signal intensity, increase were lower than in theory, the actual profit in signal intensity was again lower than for peak concentrations. This finding points to factors beyond chromatographic enrichment that influence detector response. The most polar analytes including amino acids were quantitatively lost during the loading step in the nano-setup. Detector response of the other molecules and in the micro-setup was affected (i.e., mostly reduced) by extra-column effects, for example, signal suppression (during ionization or in the C-trap) due to up-concentrated analyte and matrix, up-concentration of analytes altering adduct formation, and non-linear ESI-response (Yu et al., 2020).

$$Z_{a}=Y_{a}\text{*}60\text{* }\frac{4}{\pi *d_{a}^{2}},$$ Equation (1)

$$Y_{m}=\frac{Z_{a}}{60}\text{*}\frac{\pi *d_{m}^{2}}{4},$$ Equation (2)

$$c_{Peak}=\frac{IV*c}{Y*fwhm},$$ Equation (3)

where *Y*_*a*_ *. Y*_*m*_ = volumetric flow rate of analytical and micro-setup [mL/min]
*Z*_*a*_ *, Z*_*m*_ = linear flow velocity of analytical and micro-setup [cm/h]
*d*_*a*_ *, d*_*m*_ = column inner diameter of analytical and micro-setup [cm]
*c*_*Peak*_ = peak concentration, average conc. across whole peak volume [µg/L]
*IV*= injection volume (3 µL)
*c* = concentration of injected sample (1 μg/L)
*Y* = flow rate of respective setup [µL/min]
*Fwhm* = peak width at half maximum [min]

#### 3.2.3 Linear Range

Non-targeted exploratory -*omics* investigation typically involves fold-change analysis of signal intensities between studied sample groups and ideally, the linear range of the analytical platform should cover the range of analyte concentrations in the different sample groups to properly compare them (Alseekh et al., 2021).

Linear range was estimated for a panel of exogenous molecules according to The Fitness for Purpose of Analytical Methods: A Laboratory Guide to Method Validation and Related Topics (Eurachem, 2nd ed. 2014) and spanned between 2 and 4 orders of magnitude (Eurachem, 2014). On average, it was shorter and reached lower concentrations in the nano-setup compared to micro- and analytical flow platform (Supplementary Figure S1). Linear range length of individual molecules was affected by matrix effects in all setups. A wide linear dynamic range is advantageous in assays where the individual analyte is expected to appear in a wide concentration span, as in metabolomics, and the shorter linear range in the nano-setup arguably complicates quantitative comparison of samples or sample types that contain vastly different quantities of analyte. However, quantification of low abundant analytes profits from higher sensitivity. It depends on expected analyte concentration and goal of the analysis which of these aspects is given more importance.

#### 3.2.4 Limit of Detection

The limit of detection (LOD) estimates the concentration at which an assay can accurately predict if a compound is present in the sample or not. As such, it views signal intensity in conjunction with a level of certainty, which is represented by signal repeatability at concentrations on the verge of being undetectable. To account for possible compound-specific carryover and background and consistently select appropriately low concentrations for each platform, LOD was calculated based on relative area standard deviation of four repeated injections of the lowest standard/sample concentration in the linear range (Eq. 4). In account of the lower linear range concentrations of the nano setup (Supplementary Figure S1), the average concentration of standards used for LOD calculation were lower for the nano-setup than for the other two. LOD values averaged around 0.09 and 0.32 μg/L for standard and plasma extract in the analytical flow setup, respectively. LODs in the micro-setup reflected the gains in signal intensity (0.06 and 0.15 μg/L for standard and plasma extract, respectively). For the nano-setup, the gains in signal intensity did not equally translate to higher reproducibility of chromatographic peak area, resulting in LOD values much higher than expected judging from the high signal intensities (median values of 0.08 μg/L and 0.21 μg/L for standard and plasma extract, respectively). Drawing on the retention time, specific distributions of signal intensity and repeatability in the three setups, the actually obtainable profit regarding LOD was very much compound dependent. For some compounds finding already ideal chromatographic conditions and sufficient signal intensity in the analytical flow setup, LODs nominally even decreased upon miniaturization (Supplementary Figure S2). There are several ways to calculate the LOD and all give slightly different results, favoring systems with high signal stability over those with high signal intensity, or vice versa. At any rate, detection limits need to be understood as an estimate only. Regarding the nano-setup, we can draw from this comparison that LODs did not improve equally to signal intensity due to practical instrumental issues like carryover and impaired signal stability.

$$Limit\;of\;detection=\frac{3*sd}{slope},$$ Equation (4)

where *sd* = standard deviation of the chromatographic peak area (background corrected) of repeated injections of standard/spiked plasma extract with a concentration equal to the lowest calibration point in the linear range (*n* = 4)
*slope* = slope of calibration curve in the linear range

#### 3.2.5 Matrix Effect

**FIGURE 5:**
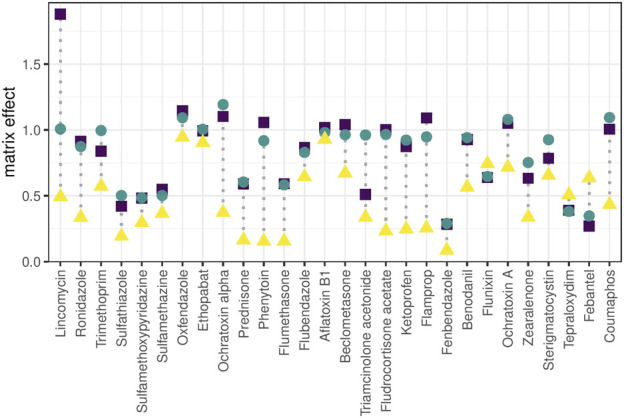
Matrix effect. Matrix effect is calculated for exogenous compounds as the calibration curve slope (linear range) obtained for spiked plasma extract relative to the slope obtained for pure solvent. Ratio <1: matrix-related ion suppression, ratio >1: matrix-related signal enhancement, ratio = 1: signal intensity was not influenced by matrix effects. Molecules are ordered from low to high retention time (left to right).

Matrix effect was calculated as the ratio of calibration curve slopes between plasma extract and standard for a panel of exogenous molecules. A crude human plasma extract was chosen as model matrix to challenge the systems with matrix complexity often encountered in *-omics* experiments of biological samples. On average, all setups showed signal suppression and the steepness of calibration curves was reduced through the matrix (Figure 5) without any obvious relation to retention time. The effect was especially notable in the nano-setup with an average sensitivity loss of almost 50% compared to matrix-free samples. The trap-and-elute configuration removed hydrophilic matrix components like salts that hamper ionization, but other matrix components were retained on the trap column and eluted in the relevant retention time window together with the targeted analytes in up-concentrated form. The nano-system showed even higher ion suppression compared to micro- and analytical setup. This is attributed to reduced chromatographic resolution due to the observed chromatogram compression. In fact, this experiment showed that the nano-system is hardly compatible with the fast and steep gradients applied in wide-target small-molecule analysis.

## 4 Discussion

For the present study we selected three platforms representing practical solutions in different *-omics* disciplines and demonstrated how different grades of LC miniaturization fundamentally affect chromatographic parameters related to successful non-targeted LC-ESI-MS-based *-omics* analysis. From a chromatographic perspective, system optimization for quantitative assays includes adjustments of separation, minimizing peak widths and maximizing signal intensity and chromatographic compound resolution (determined by retention, selectivity and efficiency) in the shortest possible time. In the application of non-target HRMS, this optimization strategy needs to be reassessed.

**FIGURE 6:**
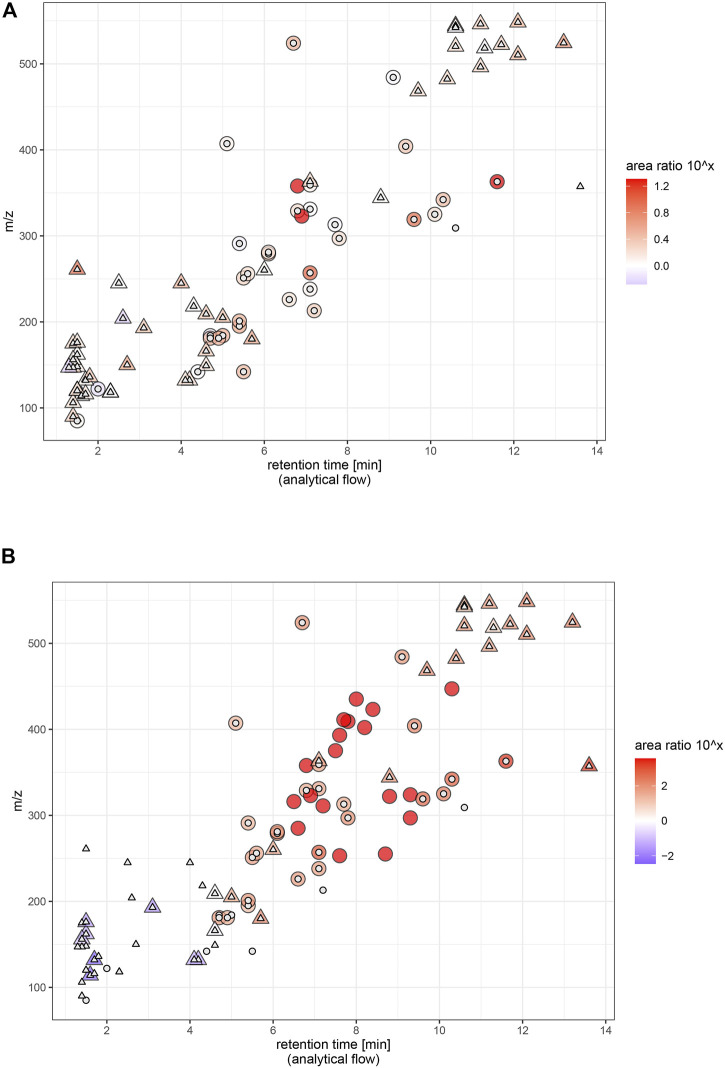
Signal intensity (relative) and physicochemical coverage. Endogenous and exogenous molecules (ceftiofur and coumaphos 10 μg/L, caffeine, paraxanthine and theobromine naturally occuring abundance, all others 1 μg/L) in plasma extract. Panel **(A)** micro, panel **(B)** nano. Ratio = area in miniaturized setup/area in analytical flow setup. Triangles represent endogenous metabolites, circles represent exogenous analytes. Large icon = molecule has been found in miniaturized setup, small icon: molecule has been found in analytical flow setup. Areas are background corrected. LPC 20:1 was excluded to facilitate visual comparison (ratio ∼6,000 in nano and ∼200 in micro).

Miniaturization to micro-flow regime on average yielded moderately increased sensitivity as expected. The flow rate for the micro-flow setup ranged in a similar magnitude as the one used for the analytical flow setup and used the same ion source. The theoretical signal intensity increase is 4.4-fold based on reduced radial dilution on column. In practice, we saw that the actual profit is largely compound-dependent, and does for most of the molecules not entirely reflect enrichment success (expressed as peak concentration). This is related to the detection process. ESI-MS does not necessarily respond with twofold intensity when analyzing a sample with twice the concentration (Patti, 2011). Rather, increasing (peak) concentration to n-fold leads to a lower than n-fold increase in signal intensity, an effect coined as fold-change compression (Yu et al., 2020). Micro-LC falls far behind nano-LC regarding sensitivity increase, but the gain comes at almost no cost: Micro-LC can be installed on the same instruments as microbore LC and thus offers equal robustness, method adaptability and ease of use. An indispensable feature for the employed workflow was facile stopping and re-starting of the eluent flow, which enabled just-in-time (offline) mass calibration and optimum mass accuracy conditions, thus exploiting the full identification selectivity of HRMS. Notably, while offering only incremental improvement of signal intensity, micro-LC-ESI-MS equals the analytical flow platform regarding chromatographic selectivity, positive-negative-switching ability, peak shape, handling of eluent compositions, and steep gradients. Micro-LC suits the chemical diversity of small molecules as much as established analytical flow platforms with slightly increased signal intensities for most of the molecules (Figure 6A) and around ¼ of the eluent consumption.

Nano-LC coupled to MS is valued for enhancing ionization (Wilm, 2011) and reducing matrix effects, which potentially allows increasing signal intensities beyond chromatographic enrichment. Exploiting the benefits of “true nano-ESI” would be a unique argument in favor of using nano-LC for *-omics* analysis and could make up for the practical complications of using a nano-platform even when sample size is not limited. In practice and comparable to the micro-flow setup, we found that signal intensities were far below the theoretical 880-fold increase derived from the large-volume injection, and we did not see more efficient ionization or reduced matrix effect. The nano-platform was configured in accordance with practical proteomics solutions to maximize robustness, whereas the benefits of “true nano-ESI” only emerge at lower flow-rates (<50 nL/min) and with narrower spray tips (outer diameter of few µm) (Schmidt et al., 2003). The hardware setup we employed offers advantageous practical handling for metabolomics analysis–the (relatively) higher flow-rates can be precisely controlled and the larger inner diameter prevents the emitter from clogging, thus enabling serial analysis of protein-precipitated samples. Signal intensity and sensitivity were multiplied for many molecules upon miniaturization to the nano-flow platform, including several compounds below LOD under micro- and analytical flow. However, the platform’s unrivaled mass sensitivity based on chromatographic enrichment is tightly connected to a specific logP and *m/z* segment and was limited for smaller (<200 Da) and more hydrophilic (logP < −0.5) metabolites of the investigated panel. Some of the more polar compounds including most amino acids were completely lost to the nano-LC investigation (Figure 6B). Additionally, the nano-platform did not allow automated positive-negative-switching as the emitter needs to be positioned manually for negative ionization mode. This is necessary to maximize sensitivity and avoid corona discharge or breakdown of the spray. The chemical range covered by one run is thus reduced and the manual adjustments compromise automatability and quantitative reproducibility. Moreover, electrospray obtained with the nano-ESI source is not as stable as with the heated ESI source when adopting a wide range of eluent compositions (1–99% (v/v) organic). Overhead times for spray stabilization forbid just-in-time offline mass calibration and create reluctance toward spontaneous method adaptations once the system is successfully running. Varying chromatographic peak shapes complicate parameter optimization for data-dependent fragmentation and peak-picking in non-targeted assays.

LC miniaturization is most promising for analyte panels with similar chemical properties, which make it possible to tailor chromatography and maximize chromatographic enrichment. As such, miniaturized chromatography has been successfully applied for highly sensitive peptidomics and lipidomics and has facilitated chemical residue analysis in environmental research (Wilson et al., 2015; Yi et al., 2017; Zardini Buzatto et al., 2020). However, is it also a viable approach for global metabolomics considering the broad diversity of metabolites? The covered physicochemical spectrum was demonstrably reduced under high degrees of miniaturization and we conclude that specificity of enrichment and the need to adapt chromatographic parameters more stringently to the compounds of interest, the problematic implementation of steep gradients and gradient extremes and the lack of positive-negative-switching capability contradict wide-spectrum small-molecule analysis with trap-and-elute nano-LC. Only by focusing specific compound classes with similar physicochemical properties or equalizing retention and ionization properties through chemical derivatization (Luo and Li, 2017), miniaturization to nano-flow regime will exert its true potential. Conversely, micro-LC offers the best compromise between improving signal intensity and metabolome coverage, despite the fact that only incremental gains can be achieved. Hence, we recommend using micro-LC for global metabolomics experiments.

## Data Availability

The original contributions presented in the study are included in the article/Supplementary Material. Further inquiries can be directed to the corresponding author.

## Supplementary Material

Supplementary Figure S1 Linear range in spiked plasma extract **(A)** and pure standard **(B)**. Instrument response was plotted against concentration and linearity was assessed by visual inspection of the resulting plot and linear regression line, supported by statistics and appropriate R^2^ values (The Fitness for Purpose of Analytical Methods: A Laboratory Guide to Method Validation and Related Topics 2014). Tested concentration range: 0.01–100 µg/L for mycotoxins, 0.1–5,000 µg/L for all other molecules. Molecules are ordered by retention time (top left: lower rt, bottom right: higher rt). Sulfonamides/sulfonate, nitrates, and thiophosphates are marked with (*).

Supplementary Figure S2 Limit of detection obtained for test compounds spiked to plasma extract based on area standard deviation of N = 4 repeated injections at the lowest concentration in the linear range. Calculation is described in the main text. Sulfonamides/sulfonate, nitrates, and thiophosphates are marked with (*).

## Abbreviations

EIC, extracted ion chromatogram; ESI, electrospray ionization; HRMS, high-resolution mass spectrometry; i.d., inner diameter; LC, liquid chromatography; LOD, limit of detection; MS, mass spectrometry; o.d., outer diameter; rsd, relative standard deviation.
