# Supplementary material for: Systematic Investigation of LC Miniaturization to Increase Sensitivity in Wide-Target LC-MS-Based Trace Bioanalysis of Small Molecules
Source: Front Mol Biosci. 2022 Jul 18;9:857505. doi: 10.3389/fmolb.2022.857505 (PMC9340153; doi:10.3389/fmolb.2022.857505)
Supplement: Supplementary file 1 [file DataSheet1.docx]

Supplementary Material

# Supplementary tables

For table S1 - S5, please see adjacent excel sheet.

Table S1 Test molecules.

Table S2 Area, peak width and retention time of molecules in standard.

Table S3 Area, peak width and retention time of molecules in spiked plasma extract including endogenous metabolites.

Table S4 Calibration curve, linear range and LOD for molecules in standard.

Table S5 Calibration curve, linear range and LOD for molecules in spiked plasma extract.

# Supplementary figures


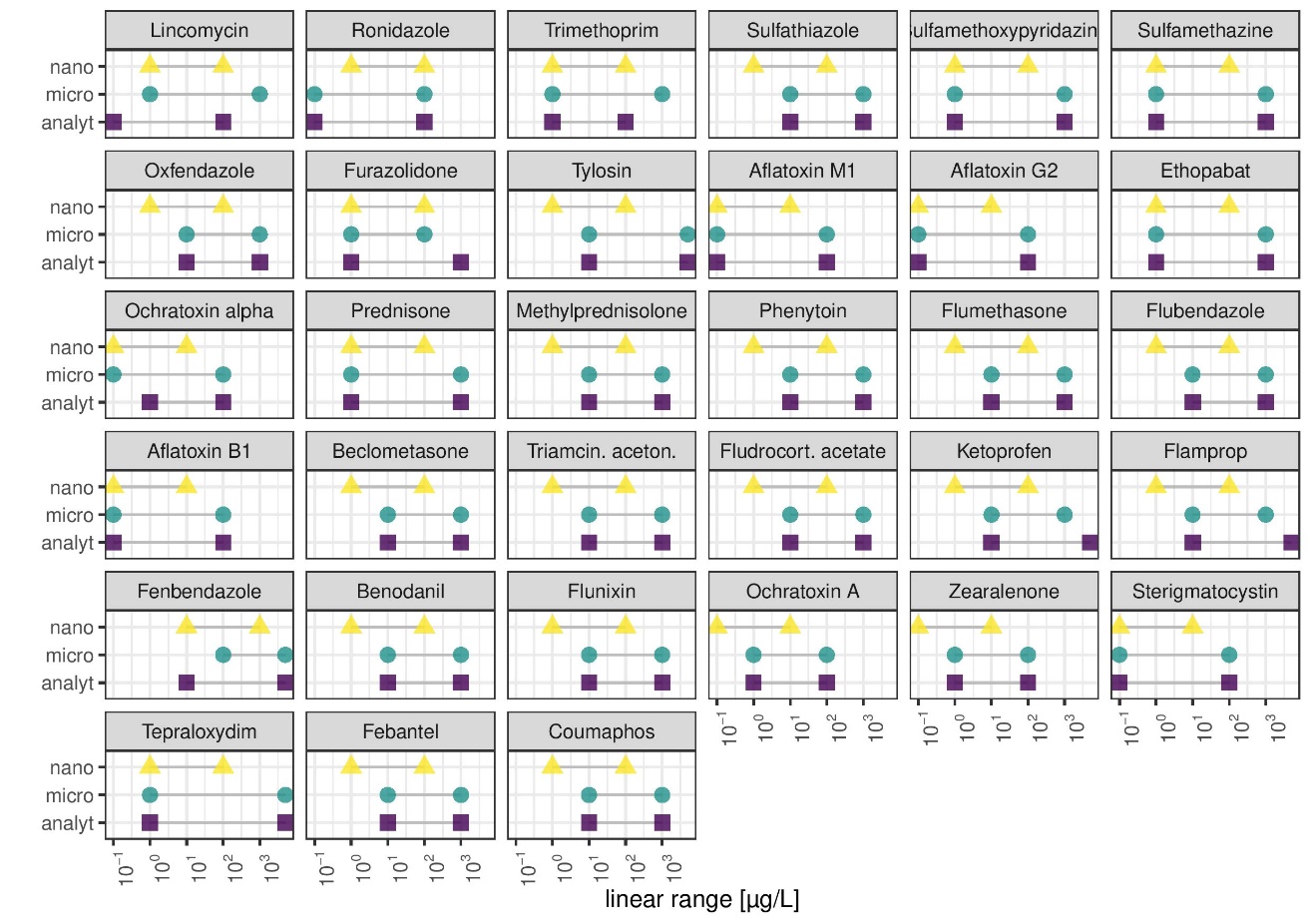


**A**


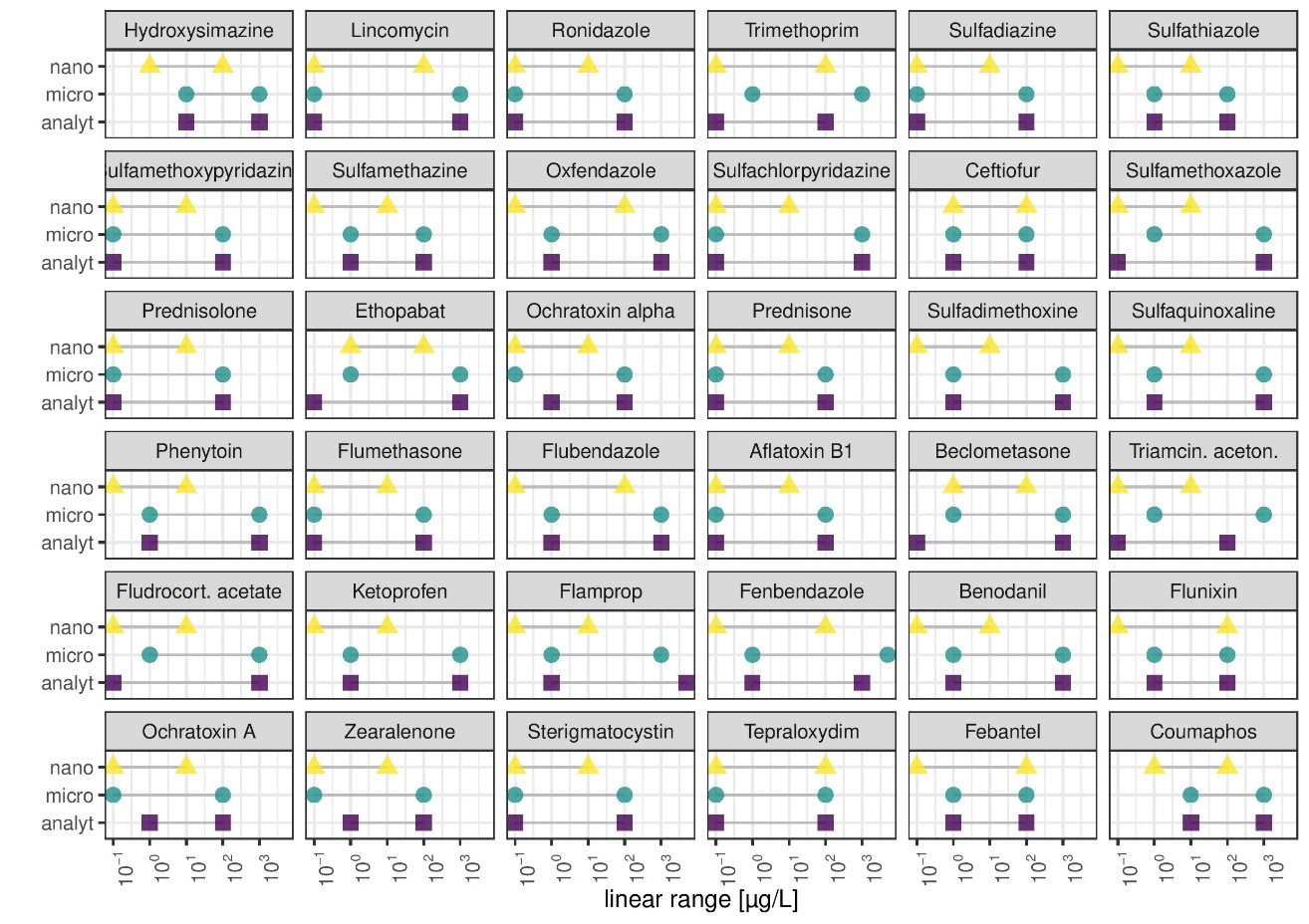


**B**

**Figure S1 Linear range** in spiked plasma extract (**A**) and pure standard (**B**). Instrument response was plotted against concentration and linearity was assessed by visual inspection of the resulting plot and linear regression line, supported by statistics and appropriate R^2^ values (*The Fitness for Purpose of Analytical Methods: A Laboratory Guide to Method Validation and Related Topics* 2014). Tested concentration range: 0.01-100 µg/L for mycotoxins, 0.1-5000 µg/L for all other molecules. Molecules are ordered by retention time (top left: lower rt, bottom right: higher rt).


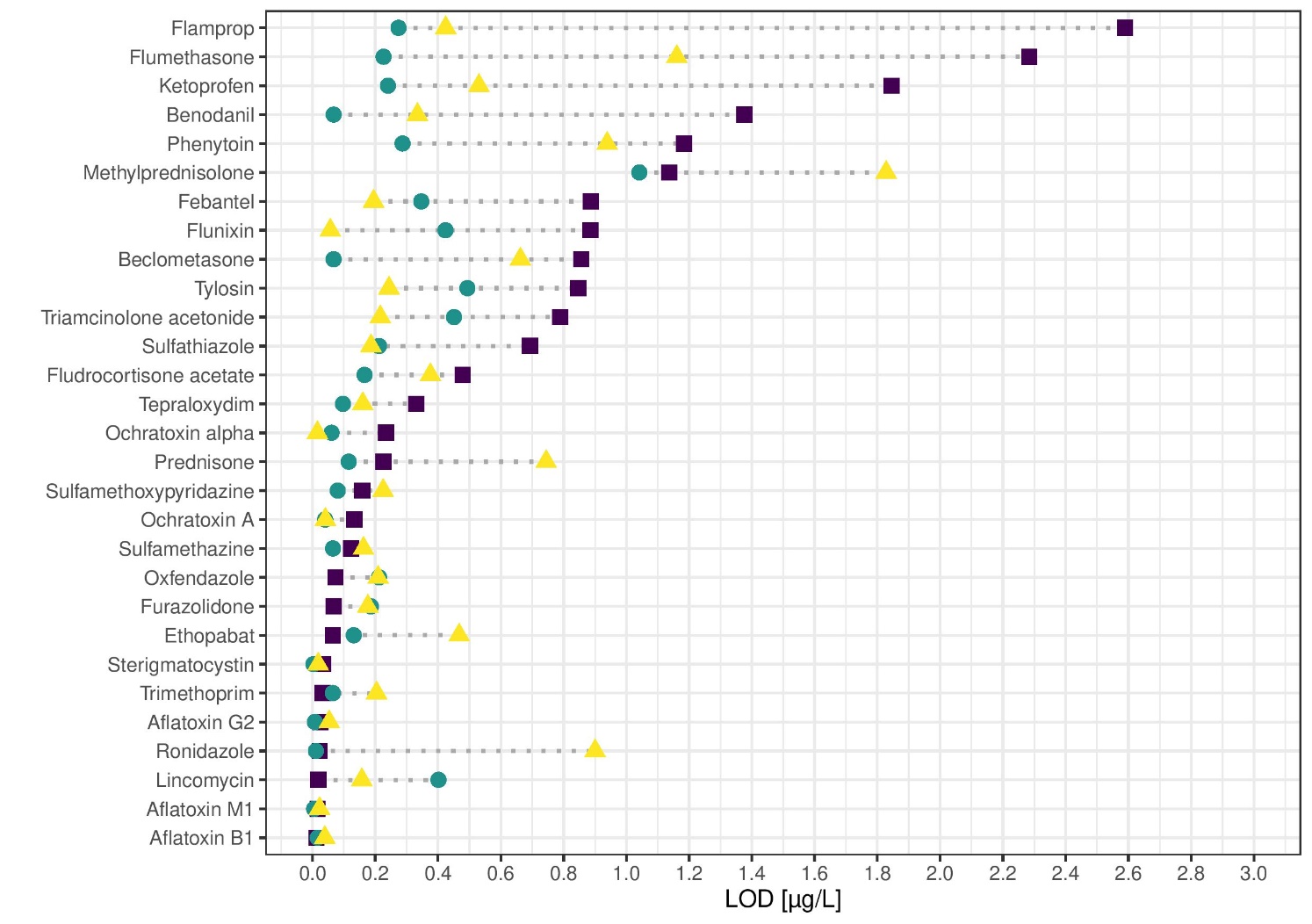


**Figure S2 Limit of detection** obtained for test compounds spiked to plasma extract based on area standard deviation of N = 4 repeated injections at the lowest concentration in the linear range. Calculation is described in the main text.
